# Immunogenicity and Safety of Sabin Strain Inactivated Poliovirus Vaccine Booster Dose Administered Separately or Concomitantly with Inactivated Hepatitis A Vaccine or Measles–Mumps–Rubella Combined Attenuated Live Vaccine: An Open-Labelled, Randomized, Controlled, Phase 4 Clinical Trial

**DOI:** 10.3390/vaccines13111087

**Published:** 2025-10-23

**Authors:** Jialei Hu, Weixiao Han, Kai Chu, Hengzhen Zhang, Ling Tuo, Xiaoqian Duan, Jun Li, Fang Yuan, Chunfang Luan, Hongxing Pan, Peng Jiao

**Affiliations:** 1 Jiangsu Provincial Center for Disease Control and Prevention (Jiangsu Provincial Academy of Preventive Medicine), Nanjing 210009, China; huctj@126.com (J.H.); chukai19812007@163.com (K.C.); 2 Sinovac Holding Group Co., Ltd., Beijing 100085, China; hanwx@sinovac.com (W.H.); zhanghz3877@sinovac.com (H.Z.); tuol8735@sinovac.com (L.T.); duanxiaoqian@sinovac.com (X.D.); lijun@sinovac.com (J.L.); yuanfang@sinovac.com (F.Y.); luancf@sinovac.com (C.L.)

**Keywords:** poliovirus, inactivated vaccine, Sabin strain, concomitant vaccination, immunogenicity, safety

## Abstract

Objectives: To determine whether a Sabin strain inactivated poliovirus booster (sIPV) given concomitantly (same day, different sites) with MMRV or inactivated hepatitis A vaccine (HepA) is non-inferior in immunogenicity and comparable in safety to separate administration at the 18-month visit. Methods: Open-label, randomized Phase IV trial in healthy children (18~22 months), allocated 2:2:2:1:1 to sIPV&MMRV, sIPV&HepA, sIPV-only, MMRV-only, or HepA-only. Primary endpoints were Day-30 seroconversion rates (SCRs) for poliovirus types 1–3 (sIPV arms) and for HepA (HepA arms). Results: Of 892 screened, 889 were randomized; baseline characteristics were balanced. By Day 30, seroprotection was 100% for PV types 1–3 in all sIPV-containing arms. SCRs were high and similar across concomitant vs. separate administration; all pairwise SCR differences met non-inferiority (−10% margin). Adjusted post-vaccination GMTs were comparable across serotypes. For HepA, Day-30 SPoR was 99.3% vs. 100.0%, and adjusted GMC was 412.2 vs. 465.9 (*p* = 0.2224). For MMRV, Day-30 SPoR was 100% in both groups with similar adjusted GMCs. Within 30 days, overall adverse reactions were 14.5%/17.4%/16.3%/7.3%/10.8% (sIPV&MMRV/sIPV&HepA/sIPV-only/MMRV-only/HepA-only), mostly mild to moderate; no vaccine-related SAEs (NCT06442449). Conclusions: Same-day sIPV co-administration with MMRV or HepA was non-inferior and well tolerated, supporting programmatic adoption.

## 1. Introduction

Since the Global Polio Eradication Initiative (GPEI) was launched in 1988, significant progress has been made in reducing the incidence of poliomyelitis worldwide. The number of cases caused by wild poliovirus (WPV) has decreased by over 99.9%, with WPV type 2 and type 3 declared eradicated in 2015 and 2019, respectively [1,2,3]. However, as of today, WPV type 1 (WPV1) remains endemic in Afghanistan and Pakistan. In 2023, these two countries reported a total of 12 WPV1 cases, compared with 22 in 2022 [4,5]. In addition to WPV, circulating vaccine-derived polioviruses (cVDPVs) have emerged as a significant concern. The number of cVDPV cases has decreased from 881 in 2022 to 524 in 2023, but the geographical spread of these cases has widened [4,6]. In 2024, cVDPV type 2 (cVDPV2) remains the predominant strain, causing paralysis in individuals across 16 countries [2,6,7]. The persistence of cVDPVs highlights the ongoing challenges in achieving global polio eradication. The World Health Organization (WHO)’s strategy is to gradually phase out OPV through introducing at least one dose of inactivated poliovirus vaccine (IPV). This measure aims to completely eliminate the occurrence of VDPV and VAPP caused by OPV vaccination [8].

In 1955, the trivalent wild-strain inactivated poliovirus vaccine (wIPV) was successfully registered and marketed in the United States. Historically, IPV supply has been concentrated among a small number of manufacturers, and as OPV was phased out and global IPV introduction accelerated, supply constraints and cost pressures emerged [9,10]. The Sabin-IPV (sIPV) is safer in production and more cost-effective compared with the wIPV. Therefore, the development of sIPV by manufacturers has been supported and encouraged by the WHO. In support of this initiative, the World Health Organization (WHO) began funding the National Institute for Public Health and the Environment in the Netherlands (RIVM), now known as Intravacc, in 2008 [11]. The goal was to develop the sIPV and to facilitate technology transfer to vaccine manufacturers in developing countries selected by the WHO. Sinovac is one of these manufacturers. Their sIPV was first approved by China’s national Medical Product Administration for marketing in 2021 and obtained the WHO pre-qualification in 2022. The safety and immunogenicity of Sinovac’s sIPV have been confirmed in multiple studies, in aspects of IPV-only regimen, sequential regimen with OPV or IPV from other manufacturers, and concomitant vaccination in its primary immunization stage with other infant routine vaccines [12,13,14,15]. In this context, this study was designed to investigate the safety and immunogenicity of co-administration of sIPV boosters with other vaccines that are specified to be given at an overlapping timing in the EPI schedule in China.

## 2. Materials and Methods

### 2.1. Study Design

This study planned to enroll 960 healthy children aged 18~22 months who had completed a three-dose sIPV primary series. With written informed consent from guardians, participants were randomized in a 2:2:2:1:1 ratio into five groups: two concomitant administration groups (C1, C2) and three separate administration groups (S1, S2, S3).

The study aimed to assess whether concomitant administration of sIPV with MMRV or HepA induces immune responses non-inferior to separate administration and maintains a comparable safety profile. Group C1 received one dose of sIPV and one dose of measles–mumps–rubella–varicella vaccine (MMRV) on the same day (sIPV&MMRV); C2 received one dose of sIPV and one dose of inactivated hepatitis A vaccine (HepA) on the same day (sIPV&HepA). S1 received sIPV alone (sIPV-only); S2 received MMRV alone (MMRV-only); and S3 received HepA alone (HepA-only). Approximately 3.0 mL of venous blood was collected from each participant 30~45 days after vaccination to measure antibody levels for immunogenicity assessment. Adverse events occurring within 30 days after vaccination were recorded. The study protocol and all participant-facing documents were approved by the Ethics Committee of the Jiangsu Provincial Center for Disease Prevention and Control (No. JSJK2024-B006-02). The trial was registered at ClinicalTrials.gov (NCT06442449) prior to enrollment and was conducted in accordance with the Declaration of Helsinki.

### 2.2. Study Population

The inclusion criteria were (1) healthy infants aged 18~22 months; (2) completion of the primary immunization with three doses of sIPV; (3) receipt of one dose of MMRV; (4) ability to provide documented proof of vaccination; (5) ability to provide legal identification; (6) the participant’s guardian being capable of understanding the study and providing written informed consent. The main exclusion criteria were (1) any vaccination with polio-containing products other than the three-dose sIPV primary series; (2) a history of two doses of MMRV, receipt of any vaccine containing measles, mumps, or rubella components, or prior vaccination against hepatitis A; (3) a history of poliomyelitis, measles, mumps, rubella, or hepatitis A; (4) a history of severe allergy to any vaccine component (e.g., anaphylaxis to streptomycin, neomycin, polymyxin B, gentamicin or gelatin, human serum albumin, formaldehyde, etc. as per package insert and WHO recommendations) [16,17]; (5) receipt of immunoglobulins or other blood products within 6 months before enrollment, or plans for such treatment during the study; (6) receipt of immunosuppressive or other immunomodulatory therapy, or cytotoxic therapy, within 6 months before enrollment, or plans for such treatment during the study; (7) receipt of a live attenuated vaccine within 14 days before enrollment, or a subunit/inactivated vaccine within 7 days before enrollment.

### 2.3. Randomization and Masking

Participants who provided written informed consent and passed screening were randomized by block allocation (block size = 16) to five arms in a 2:2:2:1:1 ratio. The randomization list was generated by an independent randomization statistician using SAS^®^ version 9.4. Eligible participants were assigned a unique randomization number (identical to the study number) in order of enrollment. To minimize the possibility of site personnel anticipating group assignments, the master randomization list was not accessible at the sites; instead, pre-numbered “scratch cards” were used. For each participant, the card corresponding to the randomization number was issued, and the allocation was revealed only after the surface coating was scratched off, ensuring allocation concealment.

### 2.4. Outcomes and Endpoints

Antibody testing was tailored to the vaccine(s) received by each group. For participants who received sIPV—i.e., the sIPV&MMRV, sIPV&HepA, and sIPV-only groups—neutralizing antibody (NAb) titers against poliovirus types 1–3 were determined using paired pre- and post-vaccination sera by microneutralization assay. For participants who received MMRV—i.e., the sIPV&MMRV and MMRV-only groups—binding antibody concentrations to measles, mumps, and rubella were measured in paired sera by enzyme-linked immunosorbent assay (ELISA). For participants who received HepA—i.e., the sIPV&HepA and HepA-only groups—binding antibodies to hepatitis A virus were quantified in paired sera using an electrochemiluminescence immunoassay (ECLIA). Laboratory testing was performed at Sinovac’s WHO-prequalified laboratory (PQ obtained 8 February 2022). The validation reports will be provided upon request. The primary endpoints were the seroconversion rates (SCRs) of neutralizing antibodies (NAbs) against poliovirus (PV) types 1–3 and SCRs of IgG to hepatitis A at Day 30 post-vaccination. Secondary immunogenicity endpoints at Day 30 included seropositivity rates (SPoRs) and geometric mean concentrations (GMCs) of IgG to hepatitis A, measles, mumps, and rubella, as well as seroprotection rates (SPrRs) and geometric mean titers (GMTs) of PV NAbs. Safety endpoints comprised adverse reactions (ARs) and serious adverse events (SAEs) occurring within 30 days after vaccination. Seroprotection against polioviruses was defined as an NAb titer of 1:8, an internationally recognized threshold. Seroconversion was defined as a change from non-protective/seropositive to seroprotective/seropositive or a ≥4-fold rise in NAb titer [18]. Seropositivity thresholds for IgG were ≥20 mIU/mL for hepatitis A, ≥200 mIU/mL for measles, ≥100 U/mL for mumps, and ≥20 IU/mL for rubella [19,20,21,22].

### 2.5. Sample Size Determination

The study tested two non-inferiority hypotheses: (a) seroconversion rates (SCRs) of neutralizing antibodies (NAb) to poliovirus (PV) types 1–3 with concomitant vaccination (sIPV + MMRV or sIPV + HepA) are non-inferior to sIPV alone; (b) the SCR of anti-HAV IgG with concomitant vaccination (sIPV + HepA) is non-inferior to HepA alone. Sample size calculations (PASS 2022) assumed an SCR of 90% at 1 month in control groups, a one-sided α = 0.025, and a non-inferiority margin of −10%. For the PV endpoints, with 1:1 allocation and 90% power, ≥205 per group were required; allowing ~15% attrition, 240 participants were set for each sIPV-involved group (sIPV + MMRV, sIPV + HepA, sIPV alone). For the HAV endpoint, using a 2:1 allocation, the HepA-only group size was set at 120, yielding >80% power. In summary: sIPV-involved groups, n = 240 each; HepA-only and MMRV-only groups, n = 120 each. As the MMRV vaccine administered in this study represents the second dose for all participants, immunogenicity endpoints related to MMRV were included solely for exploratory purposes and played no role in determining the sample size.

### 2.6. Statistical Analysis

Statistical analyses were conducted in SAS 9.4. For SCR, SPrR, and SPoR, two-sided 95% CIs were computed by the Clopper–Pearson method; between-group comparisons used chi-square or Fisher’s exact tests as appropriate. Between-group differences in SCR and their 95% CIs were estimated with the Miettinen–Nurminen method; noninferiority was assessed for SCR of antibody against poliovirus of all three serotypes and HepA); non-inferiority was concluded if the lower CI bound exceeded −10%. Antibody GMTs/GMCs were calculated on log-transformed values. Baseline (pre-vaccination) comparisons used log-scale independent-samples t-tests. Post-vaccination responses were analyzed with ANCOVA on the log scale (dependent variable: post-vaccination titer/concentration; covariate: baseline value; factor: group) to obtain adjusted GMTs/GMCs, their 95% CIs, and between-group GMT/GMC ratios with 95% CIs. The assumption of homogeneity of slopes was verified by testing the group-by-baseline interaction (*p* > 0.05), justifying the use of baseline as a covariate in the ANCOVA model. Immunogenicity was analyzed in the per-protocol set (PPS: randomized participants vaccinated and provided blood samples per protocol with valid pre- and post-vaccination assays). Safety was analyzed in the safety set (SS: all participants receiving ≥1 dose).

## 3. Results

### 3.1. Participants Disposition and Baseline Characteristics

**Figure 1:**
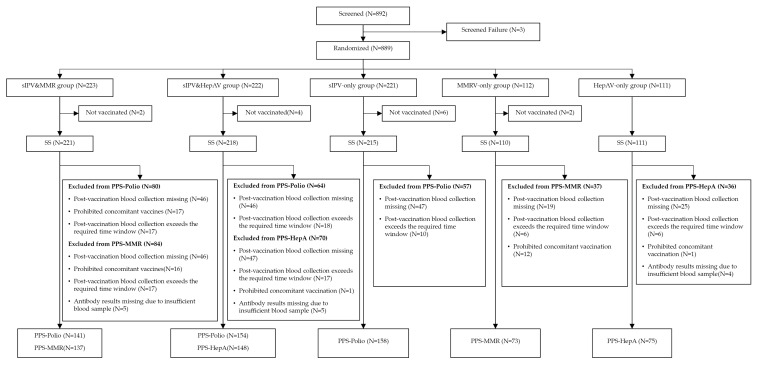
Participants Disposition. SS (Safety Set) refers to all randomized participants who received any study vaccination, analyzed for safety according to vaccine actually received. Participants who were randomized but not vaccinated were excluded from SS. PPS (Per-Protocol Set): for each antigen-specific immunogenicity analysis (PPS-Polio, PPS-MMR, PPS-HepA), the subset of SS who met the protocol and had valid paired sera collected within the prespecified window around the vaccination. Transition from SS to each PPS excluded participants with major protocol deviations relevant to that endpoint, including a. post-vaccination blood sample missing; b. sampling outside the prespecified window (30~45 days post vaccination); c. prohibited concomitant vaccination (i.e., receipt of non-study inactivated vaccines within 14 days, or attenuated live vaccines within 28 days after MMRV; or any non-study vaccine within 7 days after sIPV or HepA); d. unavailable assay results. Because PPS is endpoint-specific, a participant may be included in PPS-Polio but excluded from PPS-MMR or PPS-HepA (and vice versa) if the deviation only affects certain antigens. Minor deviations judged not to affect the endpoint did not trigger PPS exclusion. The numbers excluded from SS to each antigen-specific PPS for each reason are detailed in the figure above.

A total of 892 toddlers were screened; 889 were randomized into five groups (sIPV&MMRV, sIPV&HepA, sIPV-only, MMRV-only, HepA-only) in a 2:2:2:1:1 ratio. Study completion rates were high across groups: 220/223 (98.7%) in the sIPV&MMRV group, 218/222 (98.2%) in the sIPV&HepA group, 214/221 (96.8%) in the sIPV-only group, 109/112 (97.3%) in the MMRV-only group, and 111/111 (100.0%) in the HepA-only group, yielding an overall completion of 872/889 (98.1%). The per-protocol set (PPS) included 453 participants for poliovirus endpoints (141, 154, and 158 in the sIPV&MMRV, sIPV&HepA, and sIPV-only groups, respectively), 210 for MMR endpoints (137 in the sIPV&MMRV group; 73 in the MMRV-only group), and 223 for hepatitis A endpoints (148 in the sIPV&HepA group; 75 in the HepA-only group) (Figure 1). Baseline demographics and general examination were well balanced (*p* > 0.05). Participants were almost exclusively Han Chinese (99.5–100% across groups). The proportion of boys ranged from 48.6% to 56.4% across groups (Table 1).

### 3.2. Immunogenicity: Polioviruses

Pre-booster SPrRs and GMTs were comparable across groups for poliovirus types 1–3. By Day 30 post-booster, seroprotection reached 100% for all three serotypes in every sIPV-containing arm. Seroconversion rates (SCRs) were uniformly high and similar between concomitant and separate administration: type 1, 95.0% (sIPV&MMRV), 92.9% (sIPV&HepA), 96.2% (sIPV-only); type 2, 97.2%, 92.2%, 95.6%; type 3, 97.2%, 93.5%, 97.5%, respectively (Table 2). Pairwise absolute differences (concomitant—separate) were small and well within the prespecified non-inferiority margin (−10%): type 1, −1.2% (sIPV&MMRV vs. sIPV-only, 95%CI: −6.6%, 3.8%) and −3.3% (sIPV&HepA vs. sIPV-only, 95%CI: −9.0, 1.9); type 2, +1.6% (−3.2%, 6.4%) and −3.4% (−9.3%, 2.1%); type 3, −0.3% (−4.8%, 3.9%) and −4.0% (−9.3%, 0.7%). Non-inferiority was met for all comparisons (Table 3). Adjusted post-vaccination GMTs were likewise comparable between concomitant and separate administration across serotypes (Table 2).

### 3.3. Immunogenicity: Hepatitis A and MMR Antigens

For hepatitis A, baseline SPoR and GMCs were similar between the sIPV&HepA and HepA-only groups. By Day 30, SCR was 96.6% and 98.7% (difference: −2.1%; 95%CI: −6.6%,4.0%), SPoR was 99.3% and 100.0%, GMCs were 407.7 and 476.2 respectively, with ANCOVA-adjusted GMCs (GMCa) 412.2 vs. 465.9 (*p* = 0.2224), indicating no adverse impact of co-administration on anti–hepatitis A responses. For MMR antigens, baseline SPoR was high in both the sIPV&MMRV and MMRV-only groups. By Day 30, SPoR reached 100% for measles, mumps, and rubella in both groups. GMCa values were comparable: measles 3115.7 vs. 3268.6 (*p* = 0.4141), mumps 1697.6 vs. 2039.0 (*p* = 0.0871), and rubella 134.0 vs. 148.0 (*p* = 0.3584), indicating preserved immune responses with concomitant administration (Table 4).

### 3.4. Safety

Within 30 days post-vaccination, overall adverse reactions (ARs) occurred in 14.5% (32/221) of the sIPV&MMRV group, 17.4% (38/218) of the sIPV&HepA group, 16.3% (35/215) of the sIPV-only group, 7.3% (8/110) of the MMRV-only group, and 10.8% (12/111) of the HepA-only group. Fever was the most common systemic adverse reaction; other events—irritability, decreased appetite, diarrhea, vomiting, cough, and rhinorrhea—were also observed. Almost all ARs were mild to moderate in severity; one grade-3 AR was reported in the sIPV&HepA group. The frequency of grade-2 ARs ranged from 3.6% to 10.0% across groups (Table 5). No vaccine-related serious adverse events were reported.

Overall, concomitant administration of sIPV with MMRV or HepA elicited immune responses comparable to separate administration, achieving excellent seroprotection and maintaining a favorable safety profile.

## 4. Discussion

In this open-label, randomized Phase IV trial at the 18-month visit, same-day (different-site) coadministration of an sIPV booster with either MMRV or HepA did not compromise immunogenicity or safety compared with separate administration. For poliovirus types 1–3, SPrR was 100% across all sIPV-containing arms; non-inferiority was met for all pairwise SCR comparisons versus sIPV alone (margin −10%), and ANCOVA-adjusted GMTs were comparable by serotype. For hepatitis A, Day-30 SPoR was 99.3% and 100% in the sIPV + HepA and HepA-only groups, respectively; SCR non-inferiority was achieved and adjusted GMCs were similar. For MMR antigens, Day-30 SPoR for measles, mumps, and rubella reached 100% in both the sIPV + MMRV and MMRV-only groups, with comparable ANCOVA-adjusted GMCs. The safety profile was acceptable, with overall adverse reactions in expected ranges across groups, predominantly mild to moderate, a single grade-3 event, and no vaccine-related serious adverse events. Together, these data indicate that integrating the sIPV booster into the same visit as MMRV or HepA is immunologically sound, well tolerated, and operationally advantageous.

Our findings are consistent with prior evidence that same-day, different-site co-administration of routine pediatric vaccines preserves immunogenicity and safety. In a recent randomized study from China, concomitant sIPV + DTaP + MMRV yielded non-inferior antibody responses and similar safety compared with separate administration—mirroring our non-inferiority results for poliovirus serotypes and the absence of safety signals with sIPV given alongside measles-containing vaccines [23]. Similar conclusions were reported in preschoolers (4–6 years) where DTaP-IPV and MMRV administered together showed no adverse effect on diphtheria, tetanus, pertussis, or polio immunogenicity and were well tolerated, supporting the practicality of co-administration at booster visits [24]. Beyond high-income settings, a randomized non-inferiority trial in The Gambia demonstrated that co-administering IPV with measles–rubella and yellow fever within EPI schedules maintained immunogenicity, reinforcing generalizability across contexts [25]. Regarding hepatitis A, post-marketing evaluations indicate that inactivated HepA (Healive^®^) given concomitantly with other childhood vaccines has a safety profile comparable to HepA alone, aligning with our finding of preserved HepA responses and similar reactogenicity [26]. Finally, policy and programmatic guidance from WHO synthesize pre- and post-licensure data showing that multiple injections during a single visit are safe, effective, and operationally advantageous when schedules overlap—an evidence base our results directly support [27].

This study has several limitations that should be considered when interpreting the findings. First, immunogenicity was assessed at approximately 30 days post-booster; we did not evaluate longer-term antibody persistence, immune memory, or clinical effectiveness. Second, although the trial was adequately powered for non-inferiority on seroconversion for poliovirus types 1–3, it was not designed to detect very rare adverse events or small between-group differences in adjusted geometric mean titers; the open-label design may also have influenced subjective reactogenicity reporting despite objective laboratory endpoints. Third, generalizability may be constrained: participants were healthy 18-month-old children from a single country with predominantly Han ethnicity, and the vaccines were specific licensed products (sIPV, MMRV, and HepA) administered within one national program; extrapolation to other age groups, populations (e.g., immunocompromised or preterm children), settings, or manufacturers should be made cautiously.

## 5. Conclusions

Same-day, different-site co-administration of an sIPV booster with MMRV or inactivated HepA at the 18-month visit achieved non-inferior poliovirus seroconversion, 100% seroprotection, and preserved responses to co-administered antigens, with an acceptable safety profile and no vaccine-related SAEs. These findings support routine programmatic adoption to streamline visits and sustain coverage. Further studies evaluating long-term antibody persistence and broader population subgroups would strengthen the evidence base for expanded use.

## Figures and Tables

**Table 1:** Baseline characteristics of participants (SS).

Indicators	sIPV&MMRV Group (n= 221)	sIPV& HepA Group (n = 218)	sIPV-Only Group (n = 215)	MMRV-Only Group (n = 110)	HepA-Only Group (n = 111)	Two-Side *p* Value
Age (months)	18.2 (0.6)	18.2 (0.4)	18.2 (0.5)	18.3 (0.6)	18.3 (0.6)	0.2075
Height (cm)	82.8 (3.1)	82.7 (2.9)	82.1 (2.9)	82.6 (3.1)	82.8 (3.4)	0.0761
Weight (kg)	11.5 (1.2)	11.5 (1.3)	11.2 (1.5)	11.6 (1.3)	11.4 (1.4)	0.1116
Axillary temp	36.5 (0.3)	36.5 (0.3)	36.5 (0.3)	36.4 (0.3)	36.5 (0.3)	0.9568
Han Chinese	100%	99.5%	100%	100%	100%	0.7474
Boys	49.3%	48.6%	52.1%	56.4%	55.0%	0.6018
Girls	50.7%	51.4%	47.9%	43.6%	45.1%	

**Table 2:** Immunogenicity against polioviruses (PPS).

Indicators	sIPV&MMRV Group (n = 141)	sIPV&HepA Group (n = 154)	sIPV-Only Group (n = 158)	Two-Side *p* Value
Serotype 1				
Pre-vaccination				
Sero-protection n (%)	118 (83.7)	130 (84.4)	131 (82.9)	0.9374
95%CI	(76.5, 89.4)	(77.7, 89.8)	(76.1, 88.4)	
GMT	21.3	21.9	25.0	0.4612
95%CI	(17.6, 25.6)	(18.0, 26.6)	(20.5, 30.5)	
Post-vaccination				
Sero-protection n (%)	141 (100.0)	154 (100.0)	158 (100.0)	1.0000
95%CI	(97.4, 100.0)	(97.6, 100.0)	(97.7, 100.0)	
Seroconversion n (%)	134 (95.0)	143 (92.9)	152 (96.2)	0.4096
95%CI	(90.0, 98.0)	(87.6, 96.4)	(91.9, 98.6)	
GMT ^a^	750.5	662.7	841.6	NA
95%CI ^a^	(617.9, 911.4)	(542.7, 809.1)	(706.4, 1002.6)	
GMT ^b^	767.6	671.2	814.5	0.2892
95%CI ^b^	(639.2, 921.9)	(563.4, 799.7)	(685.0, 968.3)	
Serotype 2				
Pre-vaccination				
Sero-protection n (%)	133 (94.3)	152 (98.7)	152 (96.2)	0.1147
95%CI	(89.1, 97.5)	(95.4, 99.8)	(91.9, 98.6)	
GMT	56.3	66.5	71.7	0.2063
95%CI	(45.9, 69.2)	(55.8, 79.3)	(59.1, 87.0)	
Post-vaccination				
Sero-protection n (%)	141 (100.0)	154 (100.0)	158 (100.0)	1.0000
95%CI	(97.4, 100.0)	(97.6, 100.0)	(97.7, 100.0)	
Seroconversion n (%)	137 (97.2)	142 (92.2)	151 (95.6)	0.1380
95%CI	(92.9, 99.2)	(86.8, 95.9)	(91.1, 98.2)	
GMT ^a^	2641.9	2567.3	3074.8	NA
95%CI ^a^	(2291.9, 3045.2)	(2172.1, 3034.5)	(2671.4, 3539.0)	
GMT ^b^	2692.3	2558.5	3033.4	0.2510
95%CI ^b^	(2309.1, 3139.2)	(2209.6, 2962.6)	(2624.2, 3506.5)	
Serotype 3				
Pre-vaccination				
Sero-protection n (%)	117 (83.0)	130 (84.4)	134 (84.8)	0.9032
95%CI	(75.7, 88.8)	(77.7, 89.8)	(78.23, 90.0)	
GMT	23.2	29.1	29.7	0.2090
95%CI	(18.7, 28.7)	(23.5, 36.0)	(24.1, 36.6)	
Post-vaccination				
Sero-protection n (%)	141 (100.0)	154 (100.0)	158 (100.0)	1.0000
95%CI	(97.4, 100.0)	(97.6, 100.0)	(97.7, 100.0)	
Seroconversion n (%)	137 (97.2)	144 (93.5)	154 (97.5)	0.1422
95%CI	(92.9, 99.2)	(88.4, 96.8)	(93.7, 99.3)	
GMT ^a^	1627.8	1394.5	1851.8	NA
95%CI ^a^	(1349.2, 1964.0)	(1156.5, 1681.4)	(1559.9, 2198.3)	
GMT ^b^	1695.5	1372.8	1813.2	0.0636
95%CI ^b^	(1416.5, 2029.5)	(1156.2, 1629.9)	(1530.3, 2148.2)	

^a^ Unadjusted; ^b^ ANCOVA-adjusted. NA, Not Available.

**Table 3:** Non-inferiority of immunogenicity against polioviruses between groups (PPS).

Serotypes and Comparison Groups	SCR Difference (95%CI)	Two-Side *p* Value	One-Side *p* Value
Serotype 1			
sIPV&MMRV vs. sIPV group	−1.2% (−6.6%, 3.8%)	0.6213	0.3107
sIPV&HepA vs. sIPV group	−3.4% (−9.0%, 1.9%)	0.1931	0.0965
sIPV&MMRV vs. sIPV&HepA group	2.2% (−3.6%, 8.1%)	0.4349	0.2175
Serotype 2			
sIPV&MMRV vs. sIPV group	1.6% (−3.2%, 6.4%)	0.4650	0.2325
sIPV&HepA vs. sIPV group	−3.4% (−9.3%, 2.1%)	0.2144	0.1072
sIPV&MMRV vs. sIPV&HepA group	5.0% (−0.3%, 10.7%)	0.0605	0.0303
Serotype 3			
sIPV&MMRV vs. sIPV group	−0.3% (−4.8%, 3.9%)	1.0000	0.5741
sIPV&HepA vs. sIPV group	−4.0% (−9.3%, 0.7%)	0.0910	0.0455
sIPV&MMRV vs. sIPV&HepA group	3.7% (−1.4%, 9.1%)	0.1401	0.0700

**Table 4:** Immunogenicity against hepatitis A, measles, mumps, and rubella viruses (PPS).

Indicators	sIPV&HepA/ MMRV Group	HepA/MMRV-Only Group	Two-Side *p* Value	sIPV&HepA/ MMRV Group	HepA/MMRV-Only Group	Two-Side *p* Value
	Hepatis A			Measles		
No. of participants	148	75		137	73	
Pre-vaccination						
Sero-positivity n (%)	116 (78.4)	68 (90.7)	0.0225	134 (97.8)	72 (98.6)	1.0000
95%CI	(70.9, 84.7)	(81.7, 96.2)		(93.7, 99.6)	(92.6, 100.0)	
GMC	23.6	25.2		2025.7	1946.7	0.7892
95%CI	(22.3, 24.9)	(24.0, 26.5)	0.0749	(1746.5, 2349.6)	(1507.4, 2513.9)	
Post-vaccination						
Seroconversion n (%)	143 (96.6)	74 (98.7)	0.6663	NA	NA	
95%CI	(92.3, 98.9)	(92.8, 100.0)		NA	NA	
Sero-positivity n (%)	147 (99.3)	75 (100.0)	1.0000	137 (100.0)	73 (100.0)	1.0000
95%CI	(96.3, 100.0)	(95.2, 100.0)		(97.3, 100.0)	(95.1, 100.0)	
GMC ^a^	407.7	476.2	NA	3130.9	3238.9	NA
95%CI ^a^	(359.2, 462.6)	(417.3, 543.6)		(2862.4, 3424.6)	(2871.6, 3653.1)	
GMC ^b^	412.2	465.9	0.2224	3115.7	3268.6	0.4141
95%CI ^b^	(367.8, 462.0)	(396.9, 546.9)		(2910.8, 3335.1)	(2977.7, 3587.9)	
	Mumps			Rubella		
No. of participants	137	73		137	73	
Pre-vaccination						
Sero-positivity n (%)	115 (83.9)	58 (79.5)	0.4161	130 (94.9)	72 (98.6)	0.2665
95%CI	(76.7, 89.7)	(68.4, 88.0)		(89.8, 97.9)	(92.6, 100.0)	
GMC	259.3	237.9	0.5545	89.9	91.5	0.8558
95%CI	(218.1, 308.2)	(189.6, 298.4)		(79.1, 102.2)	(79.6, 105.2)	
Post-vaccination						
Sero-positivity n (%)	136 (99.3)	73 (100.0)	1.0000	137 (100.0)	73 (100.0)	1.0000
95%CI	(96.0, 100.0)	(95.1, 100.0)		(97.3, 100.0)	(95.1, 100.0)	
GMC ^a^	1716.7	1996.7	NA	139.7	148.6	NA
95%CI ^a^	(1476.1, 1996.4)	(1707.5, 2334.9)		(127.8, 152.7)	(134.7, 164.0)	
GMC ^b^	1697.6	2039.0	0.0871	134.0	148.0	0.3584
95%CI ^b^	(1499.9, 1921.4)	(1720.7, 2416.0)		(130.5, 150.2)	(134.4, 163.0)	

^a^ Unadjusted; ^b^ ANCOVA-adjusted. NA, Not Available.

**Table 5:** Frequency of adverse reactions developed within thirty days after vaccination (SS).

Indicators	sIPV&MMRV Group (n = 221)	sIPV&HepA Group (n = 218)	sIPV-Only Group (n = 215)	MMRV-Only Group (n = 110)	HepA-Only Group (n = 111)
Total	32 (14.5)	38 (17.4)	35 (16.3)	8 (7.3)	12 (10.8)
Grade 1	13 (5.9)	26 (11.9)	28 (13.0)	4 (3.6)	9 (8.1)
Grade 2	22 (10.0)	14 (6.4)	13 (6.1)	4 (3.6)	5 (4.5)
Grade 3	0 (0.0)	1 (0.5)	0 (0.0)	0 (0.0)	1 (0.9)
Fever	19 (8.6)	20 (9.2)	23 (10.7)	5 (4.6)	8 (7.2)
Irritability postvaccinal	3 (1.4)	6 (2.8)	9 (4.2)	1 (0.9)	2 (1.8)
Decreased activity	3 (1.4)	3 (1.4)	4 (1.9)	0 (0.0)	0 (0.0)
Vaccination site erythema	0 (0.0)	1 (0.5)	1 (0.5)	0 (0.0)	0 (0.0)
Vaccination site swelling	0 (0.0)	1 (0.5)	1 (0.5)	0 (0.0)	0 (0.0)
Diarrhea	3 (1.4)	4 (1.8)	7 (3.3)	0 (0.0)	2 (1.8)
Vomiting	1 (0.5)	3 (1.4)	5 (2.3)	1 (0.9)	3 (2.7)
Decreased appetite	5 (2.2)	1 (0.5)	8 (3.7)	1 (0.9)	2 (1.8)
Cough	3 (1.4)	1 (0.5)	2 (0.9)	0 (0.0)	2 (1.8)
Rhinorrhea	3 (1.4)	1 (0.5)	0 (0.0)	1 (0.9)	3 (2.7)
Rash erythematous	0 (0.0)	1 (0.5)	1 (0.5)	0 (0.0)	0 (0.0)
Rash	0 (0.0)	1 (0.5)	0 (0.0)	0 (0.0)	0 (0.0)
Erythema	0 (0.0)	0 (0.0)	1 (0.5)	0 (0.0)	0 (0.0)
Hypersensitivity	1 (0.5)	0 (0.0)	0 (0.0)	0 (0.0)	0 (0.0)

## Data Availability

The data presented in this study are available on request from the corresponding author.

## Abbreviations

The following abbreviations are used in this manuscript:

AE	Adverse Event
ANCOVA	Analysis of Covariance
AR	Adverse Reaction
CI	Confidence Interval
cVDPV	Circulating Vaccine-Derived Poliovirus
DTaP	Diphtheria–Tetanus–acellular Pertussis Vaccine
ECLIA	Electrochemiluminescence Immunoassay
ELISA	Enzyme-Linked Immunosorbent Assay
EPI	Expanded Programme on Immunization
GCP	Good Clinical Practice
GMC	Geometric Mean Concentration
GMT	Geometric Mean Titer
GPEI	Global Polio Eradication Initiative
HAV/HepA	Hepatitis A Virus / Inactivated Hepatitis A Vaccine*
IM	Intramuscular
IPV	Inactivated Poliovirus Vaccine
MMR	Measles–Mumps–Rubella Vaccine
MMRV	Measles–Mumps–Rubella–Varicella Vaccine
NAb	Neutralizing Antibody
OPV	Oral Poliovirus Vaccine
PPS	Per-Protocol Set
PV	Poliovirus
SAE	Serious Adverse Event
SCR	Seroconversion Rate
SC	Subcutaneous
SPoR	Seropositivity Rate
SPrR	Seroprotection Rate
SS	Safety Set
WHO	World Health Organization
wIPV/sIPV	Wild strain IPV / Sabin strain IPV
